# Effects of chronic kidney disease on complications and mortality after fracture surgery

**DOI:** 10.1186/s13741-025-00514-y

**Published:** 2025-03-22

**Authors:** Bei-Bei Lu, Xu-Rui Liu, Qing-Song Chen, Xiao-Lin Yuan, Qian Luo, Yu-Dong Hu, Xiao-Hui Liao

**Affiliations:** 1 Department of Nephrology, The Second Affiliated Hospital of Chongqing Medical University, Chongqing, 400010 China; 2 Department of Radiology, The Second Affiliated Hospital of Chongqing Medical University, Chongqing, 400010 China

**Keywords:** CKD, Fracture, Meta-analysis

## Abstract

**Purpose:**

The purpose of this meta-analysis was to evaluate the effects of CKD on postoperative complications and the survival of patients with fractures.

**Methods:**

The PubMed, Embase, Cochrane Library, and CNKI databases were searched from inception to May 15, 2024. The search strategy focused on two keywords: dialysis and hip fracture. Pooled odds ratios and mean differences were analyzed. RevMan 5.4 was used for data analysis in this meta-analysis.

**Results:**

This meta-analysis included 19 studies involving 1,615,440 patients. The CKD group had higher proportions of males, smokers, and patients with preoperative comorbidities such as diabetes, hypertension, heart failure, chronic lung disease, coronary heart disease, peripheral vascular disease, dementia, and wound infection. The CKD group also had a greater likelihood of postoperative myocardial infarction (OR = 1.67, 95% CI = 1.54–1.81, *P* < 0.00001, *I*^2^ = 33%). There was no significant difference in cerebrovascular accidents, liver failure, sepsis, and overall complications between the two groups. Additionally, the CKD group had higher mortality rates at 30 days (OR = 2.71, 95% CI = 2.23–3.28, *P* < 0.00001, *I*^2^ = 84%), 1 year (OR = 3.17, 95% CI = 2.64–3.82, *P* < 0.00001, *I*^2^ = 85%), 2 years (OR = 3.06, 95% CI = 2.88–3.25, *P* < 0.00001, *I*^2^ = 8%), and 10 years (OR = 6.85, 95% CI = 5.84–8.03, *P* < 0.00001, *I*^2^ = 0%) post-surgery compared to the non-CKD group.

**Conclusion:**

Compared with patients in the non-CKD group, patients in the CKD group did not significantly differ in the incidence of most postoperative complications after fracture surgery. However, the CKD group had a significantly greater incidence of myocardial infarction and markedly higher postoperative mortality rates at 30 days, 1 year, 2 years, and 10 years.

**Trial registration:**

PROSPERO CRD42025648208.

**Supplementary Information:**

The online version contains supplementary material available at 10.1186/s13741-025-00514-y.

## Introduction

Fractures represent a major public health concern worldwide, causing pain, disability, reduced quality of life, and substantial medical costs. They place a significant burden on families, society, and healthcare resources. While the incidence of fractures has stabilized in recent years, the absolute number of fractures has remained high, with elderly and osteoporotic fractures accounting for the majority of cases (Cauley 2021; GBD 2019). The choice of treatment for fractures typically depends on factors such as the type of fracture, the patient’s age, overall health, and functional requirements. Conservative treatments, including plaster immobilization, traction, or functional rehabilitation, are commonly employed for stable fractures or patients in poor health. Conversely, for complex fractures requiring anatomical reduction and internal fixation, surgical intervention is often the primary approach. However, surgical treatment has inherent risks, including complications such as cardiovascular events, infections, thrombosis, bleeding, and even mortality, which can significantly compromise patient health and survival (Sattui and Saag 2014; Baertl et al. 2021).

The incidence of chronic kidney disease (CKD) is increasing. The global prevalence of CKD is approximately 9.1%, meaning that approximately 700 million people worldwide suffer from this condition. The mortality rate from CKD has also risen annually, accounting for 1.4% of total global deaths. CKD is now the 12th leading cause of death globally (GBD Chronic Kidney Disease Collaboration 2020; Johansen et al. 2021). End-stage kidney disease (ESKD) is the most severe stage of CKD and is characterized by complete loss of kidney function, necessitating kidney replacement therapy such as dialysis or kidney transplantation to sustain life (Allison 2013). CKD patients often experience secondary hyperparathyroidism, calcium and phosphorus metabolism disorders, and decreased active vitamin D levels. These issues affect bone and mineral metabolism, leading to osteoporosis and significantly increasing the risk of fractures (Alem et al. 2000; Danese et al. 2006).

Previous meta-analyses have demonstrated that CKD patients have a significantly increased risk of hip and nonvertebral fractures (Vilaca et al. 2020). However, the effects of kidney function on complications and mortality following fracture surgery remain controversial. Some studies have indicated that CKD patients experience more postoperative complications and have lower survival rates than the general population does (Kim et al. 2016; Lin and Liang 2015). Conversely, other studies suggested no significant difference in postoperative survival between CKD patients and non-CKD patients (Kuo et al. 2014; Robertson et al. 2018). Therefore, this meta-analysis aimed to evaluate the effects of CKD on complications and mortality following fracture surgery.

## Methods

Our meta-analysis was conducted in accordance with the Preferred Reporting Items for Systematic Reviews and Meta-Analyses (PRISMA) statement (Moher et al. 2009). The study was registered on PROSPERO with the registration ID CRD42025648208.

### Search strategy

We searched the PubMed, Embase, Cochrane Library, and CNKI databases from inception to May 15, 2024, to identify eligible studies. The search strategy focused on two key items: fractures and CKD. To broaden the search scope, the following terms were used for “fracture": “fracture,” “bone fracture,” and “broken bones.” For "CKD," the terms included “kidney,” “dialysis,” “hemodialysis,” and “renal replacement therapy.” These terms were combined with “AND” to limit the search to titles and abstracts, and the search was restricted to studies in English and Chinese (Supplementary material 1).

### Inclusion and exclusion criteria

The inclusion criteria were as follows: (1) studies including patients with fractures; (2) studies comparing CKD and non-CKD groups; (3) studies reporting postoperative complications or mortality. The exclusion criteria were as follows: (1) studies not grouping patients by CKD status; (2) studies not reporting complications or mortality; 3) conference abstracts, reviews, letters, comments, or case reports.

### Study selection

Two reviewers independently searched the databases, removed duplicates, screened articles by title and abstract, and evaluated the full texts of the remaining articles. They determined the eligibility basis of the inclusion and exclusion criteria, resolving any disagreements through discussion with a third reviewer.

### Data collection

The following data were extracted and cross-checked by two reviewers: (1) first author, year of publication, country, study type, duration, sample size, CKD definition, and Newcastle–Ottawa Scale (NOS) score; (2) baseline characteristics such as sex, age, body mass index (BMI), and smoking status; (3) preoperative comorbidities including diabetes, hypertension, heart failure, chronic lung disease, coronary heart disease, peripheral vascular disease, dementia, and wound infection; (4) surgical details such as fracture location (femoral neck or intertrochanteric), surgical method (hip replacement or internal fixation), and length of hospital stay; (5) postoperative complications; (6) mortality.

### Outcomes and definitions

The primary outcome was postoperative mortality, defined as mortality at 30 days, 1 year, 2 years, and 10 years after fracture surgery. The secondary outcomes were postoperative complications, including cerebrovascular accidents, myocardial infarction, liver failure, sepsis, and overall complications (Wong et al. 2022).

### Quality assessment

We used the Newcastle–Ottawa Scale (NOS) to assess the quality of the included studies. The NOS is a widely used tool for evaluating the quality of nonrandomized studies, particularly cohort and case–control studies. It consists of three domains: selection of participants, comparability of study groups, and assessment of exposure or outcome. A score between 7 and 9 indicates high quality, a score between 4 and 6 indicates moderate quality, and a score below 4 indicates low quality (Stang 2010).

### Statistical analysis

In this meta-analysis, odds ratios (ORs), mean differences (MDs), and 95% confidence intervals (CIs) were calculated for dichotomous and continuous variables (Ioannidis 2008). Since the original data collected for this analysis pertained to mortality at different postoperative time points, without accounting for time-dependent risks during follow-up, ORs were used to assess the association between CKD and non-CKD groups with postoperative mortality within this time frame. The *I*^2^ test and chi-square test were used to assess statistical heterogeneity. An *I*^2^ ≥ 50% indicated high heterogeneity, suggesting the use of a random-effects model with *P* < 0.1 considered statistically significant. An *I*^2^ < 50% indicated low heterogeneity, prompting the use of a fixed-effects model with *P* < 0.05 considered statistically significant (Siddaway et al. 2019). This meta-analysis was performed via RevMan 5.4.

## Results

### Study selection

**Fig. 1:**
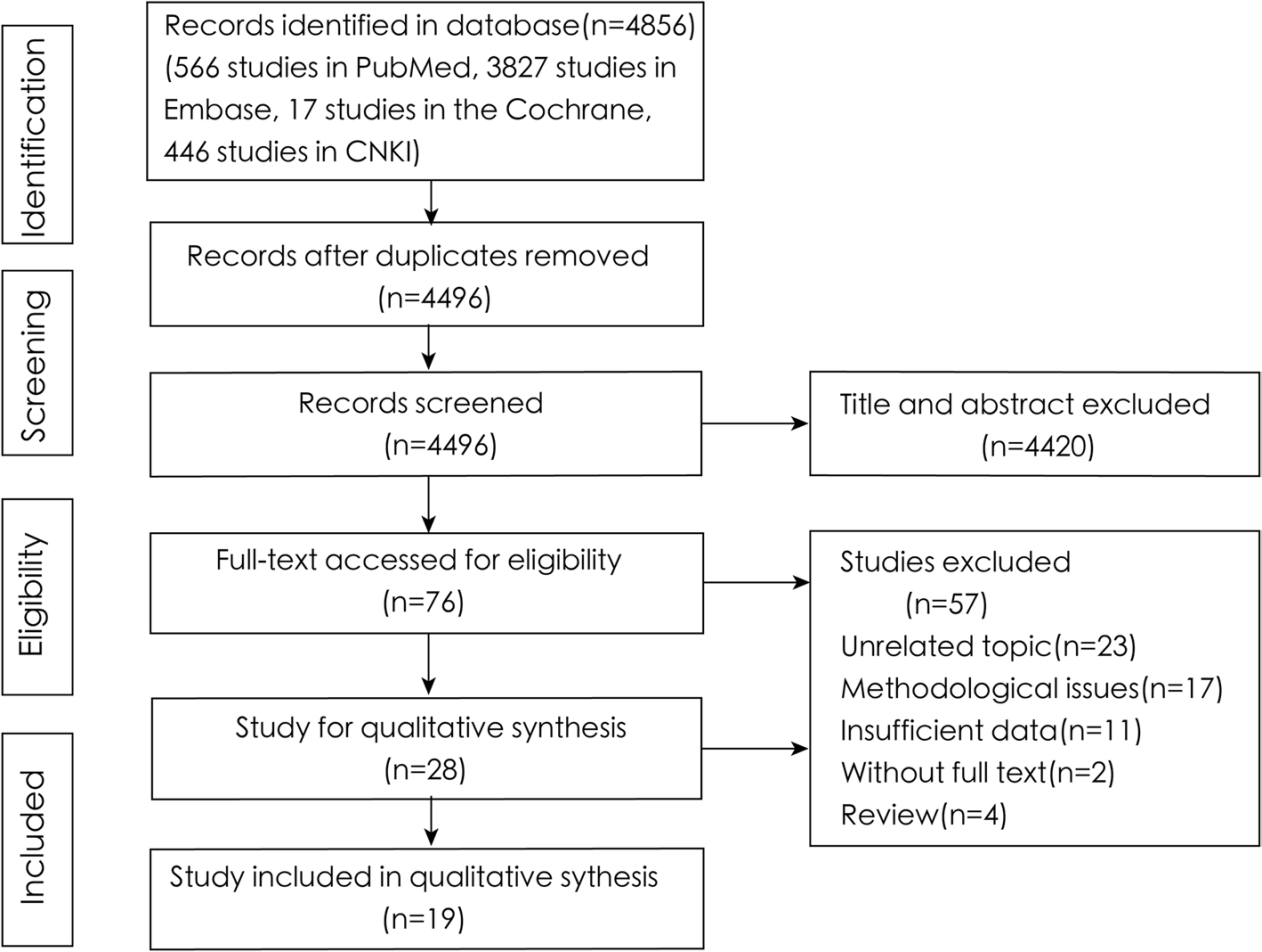
Meta-analysis

A total of 4856 articles were retrieved from the databases, including 566 from PubMed, 3827 from Embase, 17 from Cochrane, and 446 from CNKI. After 360 duplicate articles were removed, 4496 articles remained. Following a preliminary screening on the basis of titles and abstracts, 76 articles were selected for full-text evaluation. Of these, 57 articles were excluded for the following reasons: unrelated topic (*n* = 23), methodological issues (*n* = 17), insufficient data (*n* = 11), unavailable full text (*n* = 2), and review articles (*n* = 4). Ultimately, 19 articles met the inclusion and exclusion criteria and were included in this meta-analysis (Fig. 1).

### Patient characteristics and quality assessment of the included studies

Nineteen studies with a total of 1,615,440 patients were included in this meta-analysis (Kim et al. 2016; Lin and Liang 2015; Kuo et al. 2014, Robertson et al. 2018; Lee et al. 2023; Sinkler et al. 2022; Alvi et al. 2019; Puvanesarajah et al. 2018; Song et al. 2017; Ahn and Bang 2020; Jang et al. 2020; Lin et al. 2020; Hung et al. 2017; Swift et al. 2016; Maravic et al. 2014; Orabona et al. 2019; Mandai et al. 2020; Iseri et al. 2021; Blacha et al. 2009). The studies were published between 2009 and 2023. Five studies were conducted in the USA, three in South Korea, and three in Taiwan. The remaining studies were from China, the United Kingdom, France, Italy, Japan, Switzerland, and Poland. Eighteen studies were retrospective, and 1 was prospective. Sixteen studies focused on hip-related fractures, one focused on spinal fractures, one focused on ankle fractures, and one focused on fractures of the hip, spine, forearm, upper arm, or leg. The study periods ranged from 2008 to 2016. One study defined CKD as an estimated glomerular filtration rate (eGFR) < 60 ml/min/1.73 m^2^. The remaining 18 studies defined CKD as patients undergoing dialysis, four of which also included patients with eGFRs < 60 ml/min/1.73 m^2^, with two of these studies also including kidney transplant patients (Table 1). The NOS scores are presented in Supplementary Table S1.

**Table 1:** Characteristics of the studies included in the meta-analysis

					**Sample size**		**Definition of CKD**	
**Author**	Year published	Country	Study design	Study date	CKD	Non-CKD		NOS
**R. Lee**	2023	American	Retrospective	2005–2018	233	3458	Dialysis	8
**M.A.Sinkler**	2022	American	Retrospective	2005–2020	68	68	Dialysis/eGFR < 60	9
**K. Iseri**	2021	Swedish	Retrospective	2007–2016	597	151,554	Dialysis/kidney transplantation	7
**E. J. Ahn**	2020	Korean	Retrospective	2009–2015	1612	6198	Dialysis	9
**S. Y. Jang**	2020	Korean	Retrospective	2002–2015	116	16,702	Dialysis	8
**S. Mandai**	2020	Japan	Retrospective	2012–2014	9320	547,726	Hemodialysis	7
**S. J. Lin**	2020	Taiwan	Retrospective	1997–2013	9581	19,954	Dialysis/eGFR < 60	8
**N.Orabona**	2019	Italy	Prospective	2008–2016	64	64	Dialysis	7
**M. A. Alvi**	2019	American	Retrospective	2009–2016	207	73,702	Dialysis	9
**V.Puvanesarajah**	2018	American	Retrospective	2005–2014	4166	316,463	Dialysis	9
**L. Robertson**	2018	UK	Retrospective	2003–2009	19,882	19,748	Dialysis/eGFR < 60	9
**L. W. Hung**	2017	Taiwan	Retrospective	2001–2005	997	60,349	Dialysis	8
**K. S. Song**	2017	Korean	Retrospective	2009–2014	17	30	Hemodialysis	6
**S. M. Kim**	2016	American	Retrospective	2010	38,932	239,086	Dialysis/eGFR < 60/kidney transplantation	8
**O. Swift**	2016	UK	Retrospective	2009–2014	27	27	Dialysis	7
**J. C. Lin**	2015	Taiwan	Retrospective	1997–2007	2680	2680	Dialysis	9
**M. Maravic**	2014	French	Retrospective	2010	362	68,591	Dialysis	8
**L. T. Kuo**	2013	China	Retrospective	2001–2009	32	98	eGFR < 60	8
**J. Blacha**	2009	Poland	Retrospective	1996–2005	23	26	Dialysis	9

*Abbreviations*: *CKD* Chronic kidney disease, *eGFR* estimated glomerular filtration rates, ml/min/1.73 m2, *NOS* Newcastle–Ottawa Scale

### Baseline characteristics

Baseline information included sex, age, BMI, smoking status, and various comorbidities. Compared with the non-CKD group, the CKD group had a greater proportion of males (OR = 1.69, 95% CI = 1.20–2.39, *P* = 0.003) and smokers (OR = 1.51, 95% CI = 1.41–1.62, *P* < 0.00001) compared to the non-CKD group. There were no significant differences in age or BMI between the two groups. The CKD group had a greater prevalence of diabetes mellitus (OR = 2.57, 95% CI = 1.59–4.16, *P* = 0.0001), hypertension (OR = 1.74, 95% CI = 1.06–2.86, *P* = 0.03), heart failure (OR = 2.04, 95% CI = 1.48–2.80, *P* < 0.00001), chronic lung disease (OR = 1.32, 95% CI = 1.07–1.64, *P* = 0.01), coronary heart disease (OR = 2.18, 95% CI = 1.63–2.91, *P* < 0.00001), peripheral vascular disease (OR = 3.46, 95% CI = 1.25–8.28, *P* = 0.005), dementia (OR = 1.90, 95% CI = 1.05–3.44, *P* = 0.03), and preoperative wound infection (OR = 1.33, 95% CI = 1.06–1.66, *P* = 0.03) (Table 2).

**Table 2:** Summary of characteristics between the CKD group and the non-CKD group

Characteristics	Studies	Participants (CKD/ non-CKD)	Mean difference/odds ratio (95%CIs)	Heterogeneity
Baseline information				
Sex, male	17	78,029/930,915	1.69 [1.20, 2.39]; *P* = 0.003	*I*^2^ = 100%; *P* < 0.00001
Age, year	10	14,370/792,891	− 1.49 [− 3.32, 0.33]; *P* = 0.11	*I*^2^ = 99%; *P* < 0.00001
BMI, kg/m^2^	5	9625/551,338	− 0.80 [− 1.63, 0.33]; *P* = 0.06	*I*^2^ = 82%; *P* = 0.0002
Smoke	5	4706/393,789	1.51 [1.41, 1.62]; *P* < 0.00001	*I*^2^ = 39%; *P* = 0.16
Comorbidities				
Diabetes	11	57,024/1,256,884	2.57 [1.59, 4.16]; *P* = 0.0001	*I*^2^ = 100%; *P* < 0.00001
Hypertension	6	51,820/333,867	1.74 [1.06, 2.86]; *P* = 0.03	*I*^2^ = 99%; *P* < 0.00001
Heart failure	9	66,339/1,203,011	2.04 [1.48, 2.80]; *P* < 0.00001	*I*^2^ = 99%; *P* < 0.00001
Chronic pulmonary disease	6	55,954/650,232	1.32 [1.07, 1.64]; *P* = 0.01	*I*^2^ = 97%; *P* < 0.00001
Coronary heart disease	4	10,002/88,670	2.18 [1.63, 2.91]; *P* < 0.00001	*I*^2^ = 58%; *P* = 0.07
Peripheral vascular disease	3	5396/324,906	3.46 [1.25, 8.28]; *P* = 0.005	*I*^2^ = 93%; *P* < 0.00001
Dementia	3	48,875/327,631	1.90 [1.05, 3.44]; *P* = 0.03	*I*^2^ = 99%; *P* < 0.00001
Wound infection	4	10,053/43,562	1.33 [1.06, 1.66]; *P* = 0.01	*I*^2^ = 4%; *P* = 0.37
Surgery-related information				
Femoral neck fractures	4	3857/24,431	1.19 [0.95, 1.49]; *P* = 0.13	*I*^2^ = 77%; *P* = 0.005
Trochanteric fractures	4	3857/24,431	0.82 [0.63, 1.07]; *P* = 0.14	*I*^2^ = 83%; *P* = 0.0005
Arthroplasty	5	8023/340,894	1.06 [0.86, 1.31]; *P* = 0.57	*I*^2^ = 90%; *P* < 0.00001
CRIF/ORIF	5	8023/340,894	0.94 [0.76, 1.16]; *P* = 0.57	*I*^2^ = 90%; *P* < 0.00001
Length of hospital stay	4	10,227/716,515	4.41 [− 1.94, 10.76]; *P* = 0.17	*I*^2^ = 100%; *P* < 0.00001
Postoperative complications				
Cerebral vascular accident	6	5916/86,010	0.93 [0.56, 1.56]; *P* = 0.79	*I*^2^ = 92%; *P* < 0.00001
Myocardial infarction	4	12,423/34,595	1.67 [1.54, 1.81]; *P* < 0.00001	*I*^2^ = 33%; *P* = 0.21
Liver failure	5	8852/394,030	2.33 [0.63, 8.55]; *P* = 0.20	*I*^2^ = 99%; *P* < 0.00001
Sepsis	3	11,426/29,610	1.23 [0.73, 2.09]; *P* = 0.44	*I*^2^ = 75%; *P* = 0.02
Complication	4	3045/6270	1.59 [0.72, 3.54]; *P* = 0.25	*I*^2^ = 95%; *P* < 0.00001

*Abbreviations*: *CKD* Chronic kidney disease, *Cis* Confidence intervals, *BMI* Body mass index

### Surgery-related information

Surgical information included fracture location, surgical method, and length of hospital stay. There were no significant differences between the CKD and non-CKD groups regarding femoral neck fractures, intertrochanteric fractures, hip replacements, internal fixation surgeries, or hospital stays (*P* > 0.05) (Table 2).

### Postoperative complications

A comparison of postoperative complications between the CKD and non-CKD groups revealed a greater incidence of myocardial infarction in the CKD group (OR = 1.67, 95% CI = 1.54 to 1.81, *P* < 0.00001, *I*^2^ = 33%). There were no significant differences between the two groups regarding cerebrovascular accidents, liver failure, sepsis, or overall complications (*P* > 0.05) (Table 2).

### Mortality

**Fig. 2:**
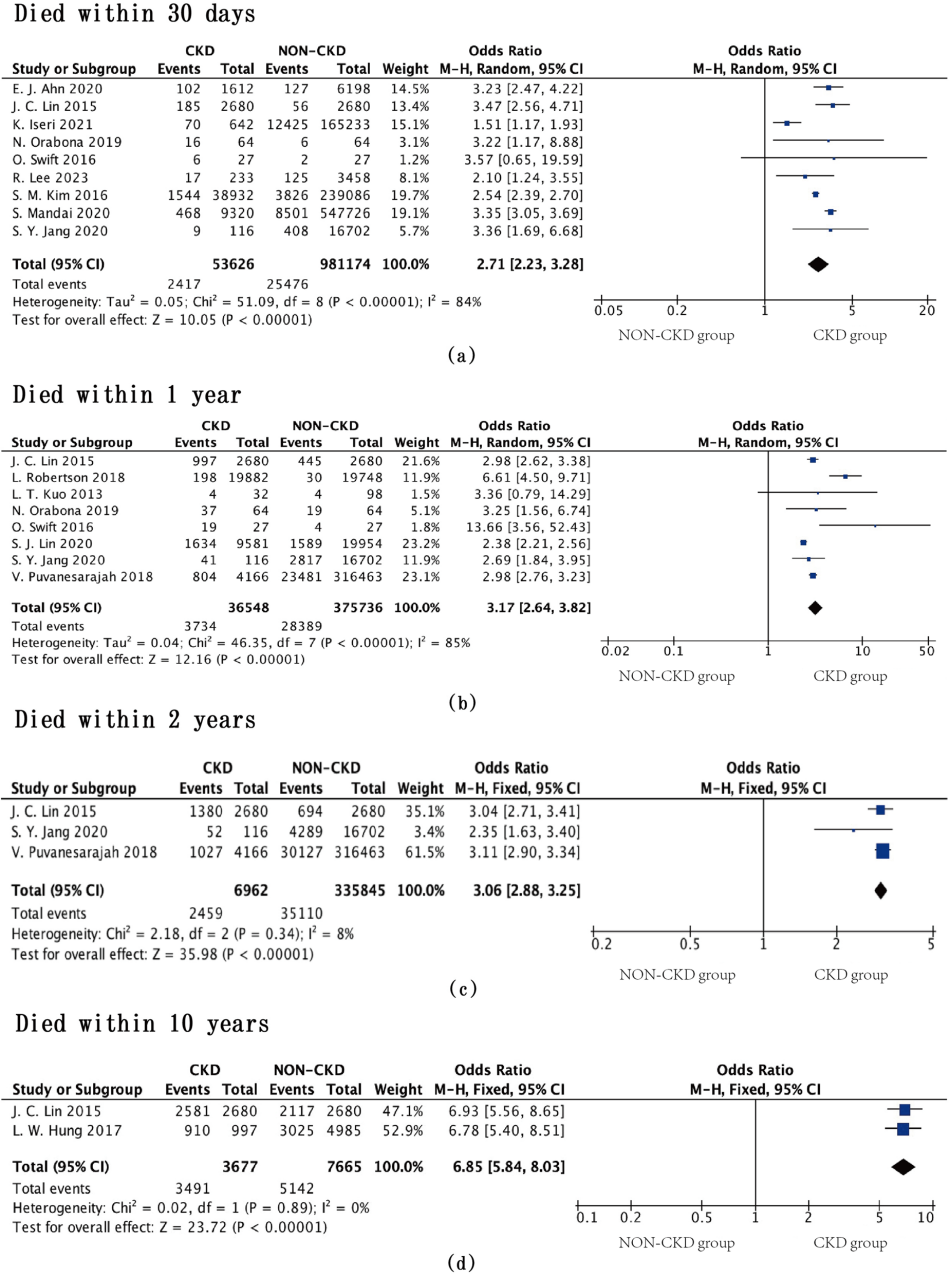
Mortality rates

We analyzed mortality at four different time points between the CKD and non-CKD groups. The CKD group had higher mortality rates at 30 days (OR = 2.71, 95% CI = 2.23–3.28, *P* < 0.00001, *I*^2^ = 84%), 1 year (OR = 3.17, 95% CI = 2.64–3.82, *P* < 0.00001, *I*^2^ = 85%), 2 years (OR = 3.06, 95% CI = 2.88–3.25, *P* < 0.00001, *I*^2^ = 8%), and 10 years post-surgery (OR = 6.85, 95% CI = 5.84–8.03, *P* < 0.00001, *I*^2^ = 0%) (Fig. 2).

### Sensitivity analysis

We performed sensitivity analyses by omitting each study one at a time. The results showed that excluding any single study did not change the overall results of the meta-analysis.

### Publication bias analysis

**Fig. 3:**
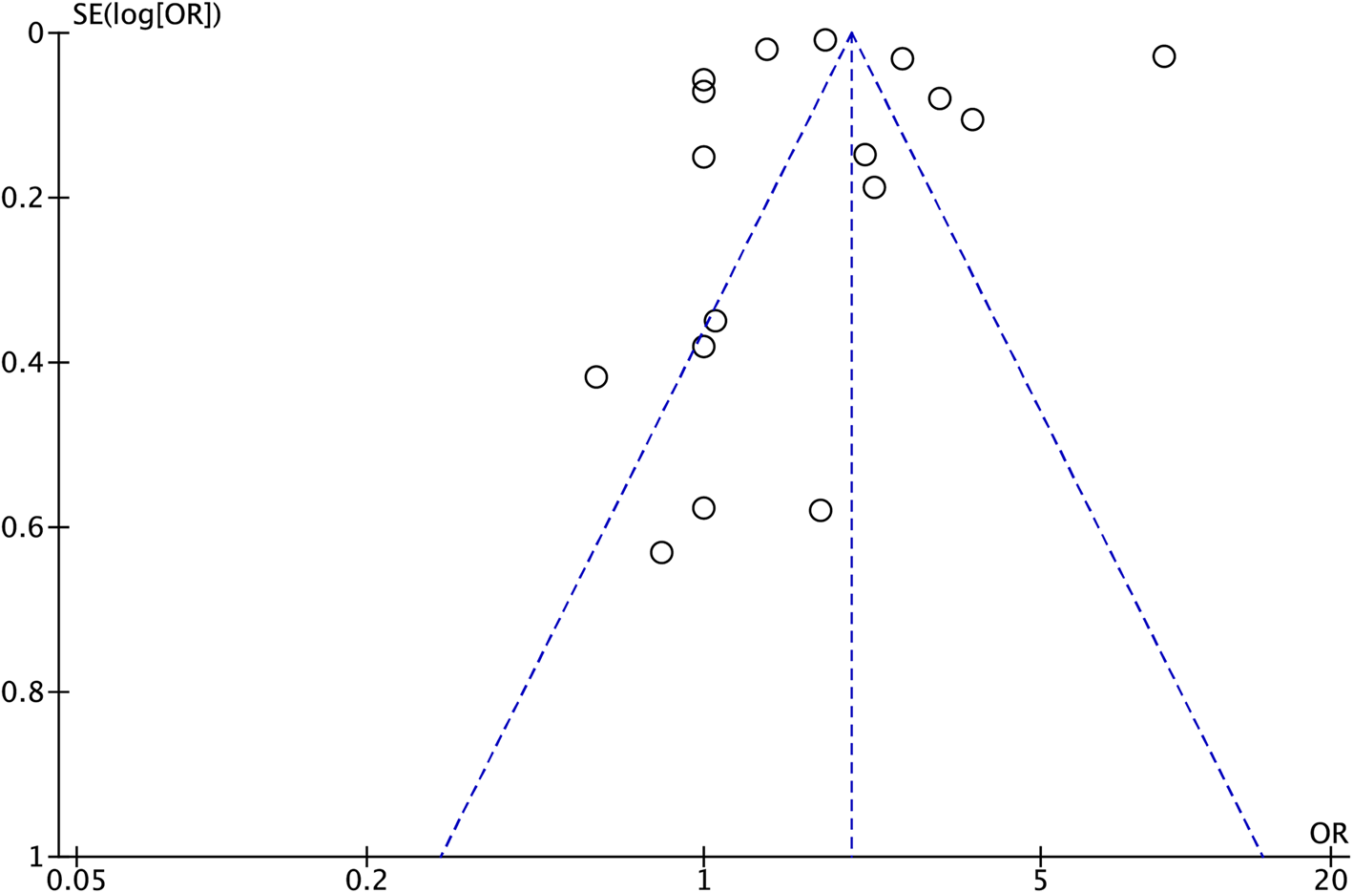
Funnel plot

A funnel plot was used to evaluate publication bias. The plot was not relatively symmetrical, and 10 plots were outside the 95% CIs, which meant that the results were affected by some publication bias (Fig. 3).

## Discussion

This meta-analysis included 19 studies involving a total of 1,615,440 patients. Each study categorized patients into CKD and non-CKD groups on the basis of whether their eGFR was < 60 ml/min/1.73 m^2^ or whether they were on dialysis. However, it is worth noting that renal transplant patients, who may not meet either of these criteria, were still included in the CKD groups in two studies (Kim et al. 2016; Iseri et al. 2021). This distinction is important for accurately interpreting the categorization and outcomes. The studies compared baseline characteristics, preoperative comorbidities, surgery-related factors, postoperative complications, and mortality between the CKD and non-CKD groups. The results revealed that the CKD group had a greater incidence of preoperative comorbidities than did the non-CKD group. With respect to postoperative complications, no significant differences were observed between the two groups for most outcomes. However, the CKD group presented a significantly greater incidence of myocardial infarction, which highlights increased cardiovascular risk in this population. Additionally, both the short-term and long-term postoperative mortality rates were significantly greater in the CKD group.

Preexisting comorbidities often have a significant effect on postoperative outcomes. Common baseline conditions include diabetes, hypertension, heart failure, and CKD (GBD Chronic Kidney Disease Collaboration 2020). These conditions may lead to increased postoperative complications and mortality. For example, poor blood sugar control in diabetic patients can affect wound healing, whereas cardiovascular diseases such as hypertension and heart failure can increase the risk of adverse events such as arrhythmias, myocardial infarction, and cerebral hemorrhage (Cram et al. 2011). Among these conditions, CKD is particularly concerning because of its complex mechanisms. Therefore, this meta-analysis aimed to provide more clinically meaningful results to guide orthopedic surgeons in managing fracture patients with CKD.

The effect of CKD on the incidence of complications after fracture surgery is still debated. Some studies reported higher complication rates in the CKD group than in the non-CKD group (Lee et al. 2023; Maravic et al. 2014), whereas others reported no significant difference (Sinkler et al. 2022; Blacha et al. 2009). This meta-analysis revealed no significant differences in the incidence of cerebrovascular accidents, liver failure, sepsis, or overall complications between the two groups. However, the incidence of myocardial infarction was significantly greater in the CKD group than in the control group, which is consistent with the findings of Kwon YE et al. (Kwon et al. 2020). This might be due to factors such as anemia, electrolyte imbalances, oxidative stress, increased myocardial load, changes in myocardial electrophysiology, atherosclerosis calcification, inflammatory responses, and hemodynamic changes in CKD patients, which collectively increase the risk of myocardial infarction (Herzog et al. 2011; K, DOQI Workgroup. 2005; Moe and Chen 2008).

The impact of CKD on mortality after fracture surgery is also inconclusive. Some studies have reported no significant difference in mortality between CKD patients and non-CKD patients (Kuo et al. 2014; Robertson et al. 2018), whereas others have reported that CKD patients have a 3–fivefold higher mortality rate after fracture than the general population (Mandai et al. 2020; Iseri et al. 2021). Previous studies have often focused on either short-term or long-term mortality, and comprehensive analyses are lacking. This meta-analysis included mortality rates at four different time points and revealed that the CKD group had significantly higher mortality rates at 30 days, 1 year, 2 years, and 10 years post-surgery. Notably, the 10-year postoperative mortality rate for CKD patients was 6.85 times greater than that for non-CKD patients. This significant difference likely reflects the natural progression of CKD and its associated complications, such as cardiovascular disease and infection, which are commonly associated with increased mortality. While surgical intervention may exacerbate certain risks, attributing such long-term outcomes solely to the procedure requires caution. A more comprehensive analysis of baseline mortality rates in CKD patients, independent of surgical intervention, is crucial to better understand the relative impact of surgery on long-term survival.

Additionally, this study analyzed several common diseases in the Charlson Comorbidity Index, which predicts morbidity and mortality after fracture surgery (Hasan et al. 2020; Schmolders et al. 2015). The incidence of diabetes, hypertension, heart failure, chronic lung disease, coronary heart disease, peripheral vascular disease, dementia, and infected wounds was greater in the CKD group than in the non-CKD group. Diabetes and hypertension are common secondary factors of CKD (Wang et al. 2023), while CKD itself increases insulin resistance and abnormal glucose metabolism, promoting diabetes (Anders et al. 2018). CKD patients also experience water and sodium retention, increasing the volume load and leading to hypertension and heart failure (Matsushita et al. 2022). CKD promotes arteriosclerosis through lipid metabolism disorders and calcium-phosphorus metabolism disorders, increasing the incidence of coronary heart disease incidence (Speer et al. 2022). Uremic neurotoxins may mediate cognitive impairment in CKD patients through interactions with neural progenitor cells, the cerebrovascular system, the lymphatic system, and monoamine neurons (Viggiano et al. 2020; Palmer et al. 2013). Toxin accumulation, renin-angiotensin system activation, increased oxidative stress, and proinflammatory cytokines increase the susceptibility of CKD patients to peripheral vascular disease and wound infection (Wu and Tarng 2020).

Anemia and malnutrition in CKD patients can prolong recovery after fracture surgery, increasing the risk of long-term complications and mortality (Hörl 2013). Therefore, it is crucial to address anemia correction and nutritional supplementation after fracture surgery for CKD patients. CKD patients are also prone to cardiovascular disease, electrolyte imbalances, and low immunity, and fracture surgery may exacerbate oxidative stress and cardiovascular load, increasing the likelihood of cardiovascular events and all-cause mortality (Foley et al. 1998; Go et al. 2004). Enhanced postoperative cardiovascular monitoring is recommended for CKD patients with cardiovascular disease. CKD patients often have osteoporosis, increasing the risk of falls and fractures. Limited mobility and prolonged bed rest postfracture increase the risk of deep vein thrombosis (DVT) and pulmonary embolism, whereas long-term inactivity can lead to muscle atrophy and further functional decline, increasing long-term mortality (Abdalbary et al. 2022). This underscores the need for special attention to postoperative rehabilitation for CKD patients.

This meta-analysis has several limitations. First, most of the included studies defined CKD patients as dialysis patients, with only five studies including patients with eGFRs < 60 ml/min/1.73 m^2^ (CKD stage 3–5). Dialysis patients often have more severe comorbidities than nondialysis patients do, placing them at an elevated risk for postoperative complications and mortality. Furthermore, different dialysis modalities, such as hemodialysis and peritoneal dialysis, may significantly impact postoperative outcomes. These variations are likely attributable to differing levels of systemic inflammation, malnutrition, and fluid imbalances commonly observed in these patient groups (Wang et al. 2020; Han et al. 2015). However, owing to limited data, differences in postoperative complications and mortality among nondialysis CKD patients, hemodialysis patients, and peritoneal dialysis patients require further study. Second, most fracture patients in this meta-analysis had hip fractures, although CKD patients often had fragility fractures of the spine, hip, radius, pubis, fibula, and proximal humerus due to bone mineral metabolism disorders (Xie et al. 2021). Future research should focus on fractures at different anatomical sites. Finally, this analysis included 18 retrospective studies and only 1 prospective study. Retrospective studies rely on existing medical records, which might lead to incomplete or inaccurate data. They could also not control for many confounding factors, potentially affecting the reliability and accuracy of the results. More prospective, multicenter, large-sample randomized controlled trials are needed to improve the reliability and comparability of the research findings.

## Conclusion

Compared with that in the non-CKD group, the incidence of most complications after fracture surgery in the CKD group was not significantly different. However, the CKD group had a significantly higher incidence of myocardial infarction and a markedly higher postoperative mortality rate. We recommend enhanced postoperative rehabilitation management and follow-up for fracture patients with CKD to improve surgical outcomes.

## Supplementary Information

Supplementary Material 1. Search strategy.

Supplementary Material 2: Table S1. Quality assessment of included observational studies using the Newcastle–Ottawa Scale.

Supplementary Material 3.

## Data Availability

The datasets used and/or analyzed during the current study are available from the corresponding author on reasonable request.
