# Supplementary material for: Effects of chronic kidney disease on complications and mortality after fracture surgery
Source: Perioper Med (Lond). 2025 Mar 22;14:34. doi: 10.1186/s13741-025-00514-y (PMC11929312; doi:10.1186/s13741-025-00514-y)
Supplement: Supplementary file 1 — Supplementary Material 1. Search strategy. [file 13741_2025_514_MOESM1_ESM.docx]

**Search Strings** *(searched in 2024/05/15)*

**PubMed: 566 Articles**

("fracture"[Title/Abstract] OR "bone fracture"[Title/Abstract] OR "broken bones"[Title/Abstract] OR "fractures, bone"[MeSH]) AND ("kidney"[Title/Abstract] OR "dialysis"[Title/Abstract] OR "hemodialysis"[Title/Abstract] OR "renal replacement therapy"[Title/Abstract] OR "chronic kidney disease"[MeSH])

**Embase: 3827 Articles**

'fracture'/exp AND ('kidney'/exp OR 'dialysis'/exp OR 'hemodialysis'/exp OR 'renal replacement therapy'/exp OR 'peritoneal dialysis'/exp OR 'estimated glomerular filtration rate'/exp)

**CNKI: 446 Articles**

(Topic: Fractures (explode)) OR (Topic: Fracture Dislocations (explode)) AND (Topic: Kidney (explode)) OR (Topic: Dialysis (explode))

**Cochrane CENTRAL: 17 Articles**

MeSH descriptor: [Fractures, Bone] explode all trees AND (MeSH descriptor: [Kidney] this term only OR MeSH descriptor: [Renal Dialysis] explode all trees OR MeSH descriptor: [Glomerular Filtration Rate] explode all trees)
