# Supplementary material for: Effects of chronic kidney disease on complications and mortality after fracture surgery
Source: Perioper Med (Lond). 2025 Mar 22;14:34. doi: 10.1186/s13741-025-00514-y (PMC11929312; doi:10.1186/s13741-025-00514-y)
Supplement: Supplementary file 2 — Supplementary Material 2: Table S1. Quality assessment of included observational studies using the Newcastle–Ottawa Scale. [file 13741_2025_514_MOESM2_ESM.docx]

**Study Quality Assessment**

**Supplementary Table:** Quality assessment of included observational studies using the Newcastle-Ottawa Scale

**Table S1.**

| Study | Year | NOS Domain | | | | | | | | | | |
| --- | --- | --- | --- | --- | --- | --- | --- | --- | --- | --- | --- | --- |
|  |  | **Selection** | | | | **Comparability** | | **Outcome/Exposure** | | | **Total Score** | **Study Quality** |
|  |  | **S1** | **S2** | **S3** | **S4** | **C1** | **C2** | **O1** | **O2** | **O3** |  |  |
| R. Lee | 2023 | ***** | ***** | ***** | ***** | ***** | ***** | ***** |  | ***** | 8 | High |
| M.A.Sinkler | 2022 | ***** | ***** | ***** | ***** | ***** | ***** | ***** | ***** | ***** | 9 | High |
| K. Iseri | 2021 | ***** |  | ***** | ***** | ***** |  | ***** | ***** | ***** | 7 | High |
| E. J. Ahn | 2020 | ***** | ***** | ***** | ***** | ***** | ***** | ***** | ***** | ***** | 9 | High |
| S. Y. Jang | 2020 | ***** |  | ***** | ***** | ***** | ***** | ***** | ***** | ***** | 8 | High |
| S. Mandai | 2020 | ***** |  | ***** | ***** | ***** | ***** | ***** | ***** |  | 7 | High |
| S. J. Lin | 2020 | ***** | ***** | ***** | ***** |  | ***** | ***** | ***** | ***** | 8 | High |
| N.Orabona | 2019 |  |  | ***** | ***** | ***** | ***** | ***** | ***** | ***** | 7 | High |
| M. A. Alvi | 2019 | ***** | ***** | ***** | ***** | ***** | ***** | ***** | ***** | ***** | 9 | High |
| V.Puvanesarajah | 2018 | ***** | ***** | ***** | ***** | ***** | ***** | ***** | ***** | ***** | 9 | High |
| L. Robertson | 2018 | ***** | ***** | ***** | ***** | ***** | ***** | ***** | ***** | ***** | 9 | High |
| L. W. Hung | 2017 | ***** | ***** | ***** | ***** | ***** |  | ***** | ***** | ***** | 8 | High |
| K. S. Song | 2017 |  | ***** |  | ***** |  | ***** | ***** | ***** | ***** | 6 | Moderate |
| S. M. Kim | 2016 | ***** | ***** | ***** | ***** | ***** | ***** | ***** | ***** |  | 8 | High |
| O. Swift | 2016 |  | ***** | ***** | ***** |  | ***** | ***** | ***** | ***** | 7 | High |
| J. C. Lin | 2015 | ***** | ***** | ***** | ***** | ***** | ***** | ***** | ***** | ***** | 9 | High |
| M. Maravic | 2014 | * | * | * | * | * |  | * | * | * | 8 | High |
| L. T. Kuo | 2013 | * |  | * | * | * | * | * | * | * | 8 | High |
| J. Blacha | 2009 | * | * | * | * | * | * | * | * | * | 9 | High |
